# Population-Level Immunity for Transient Suppression of COVID-19 Waves in Japan from April 2021 to September 2022

**DOI:** 10.3390/vaccines11091457

**Published:** 2023-09-04

**Authors:** Sachiko Kodera, Haruto Ueta, Tatsuo Unemi, Taisuke Nakata, Akimasa Hirata

**Affiliations:** 1 Center of Biomedical Physics and Information Technology, Nagoya Institute of Technology, Nagoya 466-8555, Japan; 2 Department of Electrical and Mechanical Engineering, Nagoya Institute of Technology, Nagoya 466-8555, Japan; h.ueta.106@nitech.jp; 3 Glycan and Life Systems Integration Center, Soka University, Tokyo 192-8577, Japan; unemi@soka.ac.jp; 4 Graduate School of Economics, University of Tokyo, Tokyo 113-0033, Japan; 5 Graduate School of Public Policy, University of Tokyo, Tokyo 113-0033, Japan

**Keywords:** asymptomatic infections, herd immunity, machine learning, Olympic Games, transient suppression, vaccination effectiveness

## Abstract

Multiple COVID-19 waves have been observed worldwide, with varying numbers of positive cases. Population-level immunity can partly explain a transient suppression of epidemic waves, including immunity acquired after vaccination strategies. In this study, we aimed to estimate population-level immunity in 47 Japanese prefectures during the three waves from April 2021 to September 2022. For each wave, characterized by the predominant variants, namely, Delta, Omicron, and BA.5, the estimated rates of population-level immunity in the 10–64-years age group, wherein the most positive cases were observed, were 20%, 35%, and 45%, respectively. The number of infected cases in the BA.5 wave was inversely associated with the vaccination rates for the second and third injections. We employed machine learning to replicate positive cases in three Japanese prefectures to validate the reliability of our model for population-level immunity. Using interpolation based on machine learning, we estimated the impact of behavioral factors and vaccination on the fifth wave of new positive cases that occurred during the Tokyo 2020 Olympic Games. Our computational results highlighted the critical role of population-level immunity, such as vaccination, in infection suppression. These findings underscore the importance of estimating and monitoring population-level immunity to predict the number of infected cases in future waves. Such estimations that combine numerical derivation and machine learning are of utmost significance for effective management of medical resources, including the vaccination strategy.

## 1. Introduction

Since its emergence at the end of 2019, SARS-CoV-2 has spread rapidly worldwide, leading to many hospitalizations and deaths [1]. The transmission of the virus was influenced by variants and sublineages that exhibit varying levels of infectivity and by public behavior that is influenced by factors like policies, culture, and sentiment, which vary across regions and countries [2,3]. In general, countries in the Asia–Pacific area (e.g., Hong Kong, Japan, New Zealand, Singapore, and South Korea) tended to implement stringent policies for human behavior [4] compared with European and American countries [5,6].

The spread and decay periods of the COVID-19 waves varied across countries [7]. During its first wave, China experienced a rapid decline in positive cases due to strict lockdown measures [8,9]. Similarly, Japan observed a swift spread and decline in the first wave, attributed to implementing a state of emergency [10]. In contrast, the decay periods of waves in European countries, such as the United Kingdom and Germany, were longer than that of Asian countries [7]. The spread of infectious diseases is influenced by immunity, especially adaptive immunity. Hereafter, immunity has been defined as the level of antibody-mediated protection against symptomatic infection, considering vaccine-induced immunity and naturally acquired immunity by infection. The adaptive immunity declines over time following infection and vaccination [11]. Table 1 in [12] provides a summary of the population-level immunity in various countries as of early 2021. Transient suppression, rather than herd immunity, has been suggested [13], highlighting the importance of small-scale discussions as opposed to focusing solely on country-wide approaches.

Vaccination started in December 2020 in Israel, where an abrupt drop in positive cases was observed in January 2021 [14]. The effectiveness of mRNA vaccines against symptomatic infection with the Delta variant was over 90% after two weeks of full vaccination [15,16,17,18]. However, their effectiveness against the Omicron and its subvariants was diminished due to immune evasion properties [16,19,20]. Additionally, several countries, including Taiwan, Australia, South Korea, and Japan [1,21], saw a dramatic increase in the number of new cases beginning in late 2021 or early 2022, primarily due to the emergence of viral variants with greater infectivity and shorter generation times than conventional variants [22,23]. Thus, the spatiotemporal variation in population-level (adaptive) immunity must be monitored to estimate viral transmission.

In Japan, the SARS-CoV-2 pandemic began in early 2020, and the waves were referred to as the first through seventh waves based on the cycle of reported daily positive cases (DPCs) [24]. The dominant variants during each wave were the Wuhan strain between the first and third waves, the Alpha variant in the fourth wave, the Delta variant in the fifth wave, Omicron BA.1 and BA.2 in the sixth wave, and Omicron BA.5 in the seventh wave (see Section 2.2). Specifically, prior to the seventh wave, the proportion of total reported positive cases relative to the entire population was limited to 11% (see Section 2.1), and the vaccination campaign was uniformly rolled out nationwide in March 2021. These factors contributed to the relative homogeneity of the health insurance and healthcare system, which prevented a breakdown of medical resources during the pandemic. By the fourth and fifth waves, the total number of positive cases in Japan had reached approximately 785,000 and 1,750,000, representing 0.63% and 1.4%, respectively, of the entire Japanese population.

A serologic survey conducted in Tokyo estimated that the number of infected cases was 3.9 times (95% CI: 3.0–7.0 times) higher than the reported positive cases reported [25]. Even when accounting for asymptomatic infections, the number of infected individuals is below the threshold for herd immunity that is estimated to be 50–80% [11,26,27]. Therefore, the decline observed until the fourth wave, when vaccination rates were still low, can be attributed to a reduction in the effective reproductive number due to behavioral changes prompted by the state of emergency, such as that imposed in Tokyo from 25 April 2021 to 20 June 2021, during the fourth wave [3].

The administration of the third vaccination (boost dose) began in Japan in January 2022, coinciding with the emergence of the Omicron BA.1 variant. Unlike previous waves, the sixth and seventh waves affected a large proportion of the population, with the Omicron BA.1 variant and its BA.5 subvariant being dominant, respectively. Assessing transient suppression in the 47 Japanese prefectures and applying the estimated parameters in the transmission models would provide valuable insights for future policy formulation and interventions in other regions [28].

This study aims to estimate population-level immunity during the fifth to seventh waves of COVID-19 in all 47 Japanese prefectures, particularly considering the status of vaccination and its waning effects. In particular, we analyze the temporal dynamics of population-level immunity in three metropolitan prefectures, namely, Tokyo, Osaka, and Aichi, to gain insights into wave propagation and decline timings. To validate the numerical estimate for the temporal evolution of population-level immunity, we replicated DPCs in these three prefectures using machine learning. Following the validation, we discussed the transient suppression of the fifth wave, wherein the Olympics Tokyo 2020 (summer 2021) was replicated to demonstrate the importance of monitoring population-level immunity.

## 2. Materials and Methods

### 2.1. Number of Reported Positive Cases per Day

The DPC data for each prefecture were obtained from open data provided by the Ministry of Health, Labor and Welfare [29]. The number of DPCs is influenced by factors like the incubation period and the day of the week, with typically fewer tests conducted on weekends than on weekdays. To reduce the impact of these potential variations, we employed a 7-day moving average (±3 days from the corresponding day) to smooth the data. Using the 7-day moving average of the DPCs, we determined the peak date as the day on which the highest value for each wave was observed.

**Figure A1:**
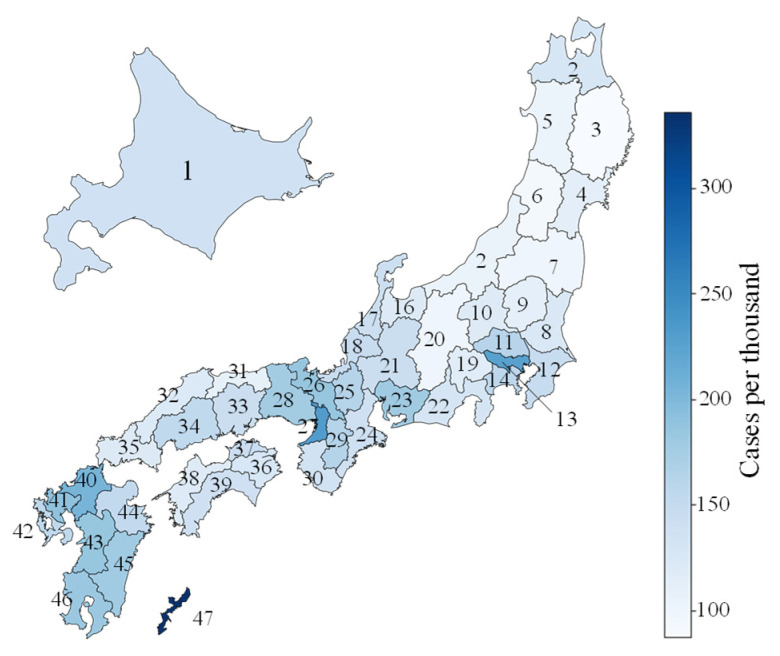
The number of positive cases reported per one thousand until the end of September 2022 in each prefecture of Japan. Each prefecture’s number corresponds to Table 2.

We assumed that each wave followed a Gaussian distribution and fitted it numerically using the least squares method to analyze the time course of the 7-day average DPCs. On the basis of the fitted curve, we defined the beginning of a wave as the date when the curve’s peak value reached 10%. In some prefectures, the decay phase of an earlier wave and the propagation phase of a subsequent wave may overlap. Notably, the DPCs remained nearly constant throughout the sixth wave’s decay phase. Table 1 presents the periods covered by the fifth to seventh waves. Table 2 shows the population density and number of reported DPCs in Japan’s prefectures from the beginning to the peak of each wave (see also Figure A1).

### 2.2. Effectiveness of Individual Vaccination and Natural Immunity

The daily number of individuals who were vaccinated (first to fourth doses) was extracted from the Vaccination Record System of the Digital Agency, Japan [30]. Vaccination in Japan first began in March 2021 for medical workers, in April for older people, and in June for nonmedical workers; vaccinations were administered nearly uniformly across the country. The interval between the first and second doses was controlled for 3 to 4 weeks. The third and fourth shots started on 1 December 2021 and 25 May 2022, respectively.

Individual vaccination effectiveness (VE) was defined as VE = 1 − relative risk in the real world without controlling for conditions, as follows:

(1) $$e_{t}\left(i\right)=\left\{\begin{array}{c} a_{t}\cdot i/K\;\left(i\leq K\right),\\ a_{t}-s\left(i-K\right)\;\left(i>K\right), \end{array}\right.$$

and

(2) $$e_{inf}\left(i\right)=a_{inf}-s\cdot i,\;$$

where *e_t_*(*i*) is the effectiveness of vaccination for an individual on day *i* after the *t*-th vaccination (*t* = 1, 2, 3, 4). This linear equation [18] is based on the logistic relationship between the neutralization level and protective efficacy [31]. This assumption agrees with a cohort study of VE in Japan [15].

The effectiveness of vaccination for an individual reaches its peak, *a_t_*, *K* days (*K* = 14 for the second dose, *K* = 7 for the third and fourth doses) after vaccination. The parameters *a_t_* for each variant were derived by comparing the prevalence of COVID-19 among unvaccinated people and without immunity to prevent infection in our previous study [28]. The waning immunity *s* by vaccination was approximated linearly. The waning effect of VE in terms of protection against symptomatic infection *s* was approximated linearly. The function *e_inf_* (*i*) denotes the natural immunity of the individual on day *i* after infection that wanes as day *i* progresses from the peak *a_inf_*. The parameters *a_t_* for each variant, *a_inf_*, and *s* were used the same as in Table 1 of [32]. The model of VE was validated in our previous studies [18,23,28,32].

The reported number of positive cases was influenced by latency, a combination of the incubation time and the time of delay in reports from hospitals/clinics to the prefecture. When considering the correlation of mobility and the effective reproduction number with positive cases, latency (the lag between reported social behavior and reported number of DPCs) was estimated at 10 and 8 days for the Delta [3] variant and the Omicron variant as well as its subvariants, respectively. After determining the peak date of the DPC, population-level immunity from 8 to 10 days prior to that day is used for appropriate evaluation.

### 2.3. Estimation of Population-Level Immunity

Population-level immunity is defined as the combined effectiveness of vaccination at the population level and the estimated proportion of infected cases among the entire population. In a serological survey conducted in Tokyo during the third wave (end of March 2021), the number of infected cases was estimated to be 3.9 times (95% CI: 3.0–7.0 times) higher than the number of positive cases [25].

As part of surveillance efforts in Tokyo from 1 April 2021, polymerase chain reaction (PCR) tests were conducted on asymptomatic individuals in downtown areas, restaurants, airports, and major train stations to estimate the population with asymptomatic infections [33].

Population-level immunity *E* is defined as the combined VE at the population level and the estimated ratio of infected cases among the entire population:

(3) $$\begin{array}{cc} E\left(d\right)=\sum_{t=1}^{T}\sum_{i=0}^{d}\sum_{s} & \left(N_{t}\left(d-i\right)\cdot e_{t}\left(i\right)v_{s}\left(d\right)\right)/P\\ + & \sum_{i=0}^{d}\sum_{s}\left(\left(1+f\right)\cdot DPC\left(d-i\right)v_{s}\left(d\right)\cdot e_{inf}\left(i\right)\right)/P. \end{array}$$

In Equation (3), the first term on the right panel represents the effectiveness of vaccination at the population level. The second term represents the natural immunity resulting from infections, assuming that natural immunity is effective only against the same variant. In Equation (3), *d* denotes the day index, *N_t_* refers to the number of individuals newly administered a vaccination dose (*t* = 1–4), *P* represents the population per prefecture (Table 1), and *v_s_* represents the rate of each variant of SARS-CoV-2 on the *d* day. It can be derived from Equations (1) and (2) considering the population of the prefectures and the waning effect of vaccination. The DPC represents the number of cases reported on a given day, and parameter *f* denotes the rate of asymptomatic individuals, assumed to be four to eight times.

The entire population and the population aged 10–64 years were considered in terms of age. The percentage of the population aged 10–64 years who acquired infection was 91%, 79%, and 81% in the fifth, sixth, and seventh waves, respectively (Figure A3); thus, this metric would potentially be related to the number of cases.

### 2.4. Replication with Machine Learning

Mathematical models such as the susceptible, exposed, infectious, and recovered (SIR/SEIR) models, Refs. [34,35,36], and deep neural networks [37] have been proposed to forecast new positive cases. However, the forecast accuracy relied on the quality of the data used for calibration. During a state of emergency, the suppression of contact makes modeling and parameter extraction more difficult. In some countries where the restriction measure had not been implemented, the SIR model was effective. However, under the restriction measure, the SIR model suffered from choosing human contact and vaccination-related parameters.

Our previous studies proposed deep neural networks, comprising a multipath neural network with long short-term memory and fully connected layers [28]. This method allowed us to understand the correlation between various factors and the incidence of infections and death cases. The training process involved using input values for 14 days along with the target output value for the next 14 days. The cross-entropy loss function was minimized using the Adam algorithm for 1000 epochs, and the training was conducted on a workstation with specific hardware specifications.

Deep learning models are often considered black boxes, but mathematically, efforts have been made to derive population-level immunity (see Section 2.3). The input values were selected based on their correlation with morbidity, and the DPCs were the desired output. In mid- and long-term projections, mobility, tweets containing risk keywords, population VE, and variant infectivity were considered, whereas meteorological data and the correlation between viral transmission and human behavior were marginal. Although this model has been primarily used for forecasting, it was also used for interpolation to estimate the impact of human behavior on new DPCs.

## 3. Results

### 3.1. Time Course of Vaccination Rate and Varriants in Japan

**Figure 1:**
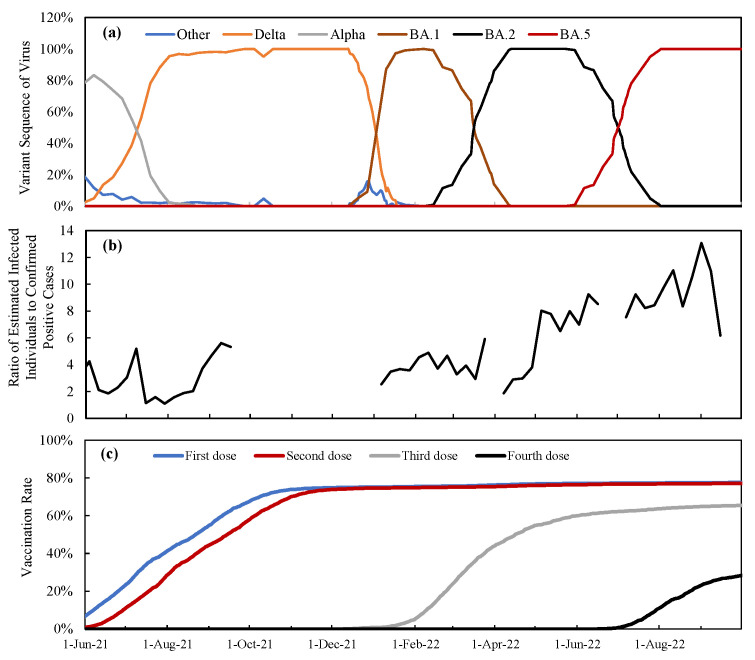
Time course of (**a**) dominant viral variants, (**b**) ratio of infected individuals without symptoms to empirically derived from the report in Tokyo, and (**c**) vaccination rate in Tokyo. In (**b**), the data for periods with less than 500 DPCs are excluded due to considerable uncertainty.

The SARS-CoV-2 sequences by variants are shown in Figure 1a. Figure 1b depicts the time course of the ratio of estimated infected cases to confirmed positive cases based on PCR tests. From the figure, the ratio varies depending on the time window that also holds true for other variants [32]. This ratio becomes relatively unstable when the number of DPCs is small. It should be noted that different operators conducted surveillance before and after April 2022. Hence, it is empirically assumed that the number of infected cases is the number of reported cases multiplied by a factor of 6 (3–5 for the Omicron variant and 7–11 for the BA.5 variant). Due to the lack of similar surveys in other regions, this assumption is applied uniformly across all prefectures. Note that this ratio is close to 3.0–7.0 times (95% CI) that obtained in earlier waves of the cohort study [25], although this ratio would vary for different age groups and viral variants. Figure 1c illustrates the vaccination rate in Japan over time. More detailed time course of vaccination in each prefecture can be found in Video S1.

### 3.2. Daily Reported Positive Cases and Estimated Population-Level Immunity

**Figure 2:**
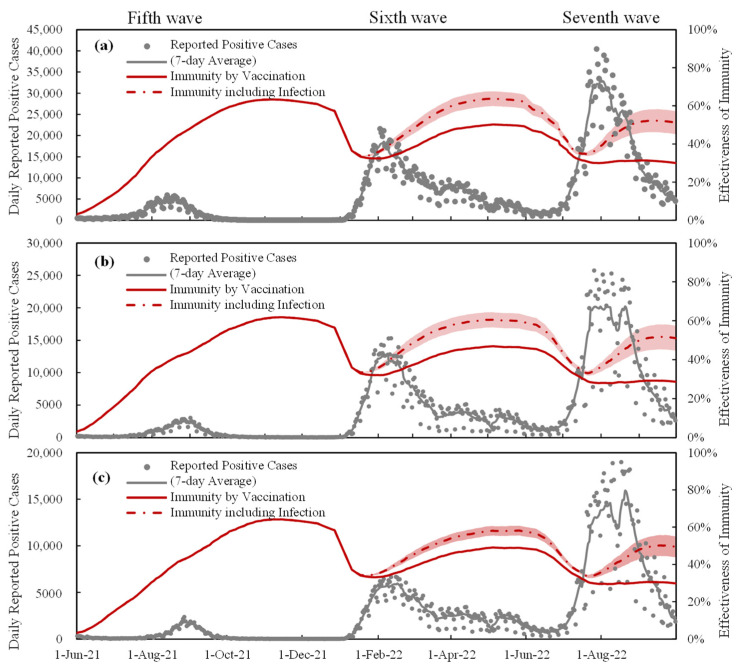
Daily reported positive cases and estimated population-level immunity in (**a**) Tokyo, (**b**) Osaka, and (**c**) Aichi. The dashed line indicates population-level immunity assuming that the number of asymptomatically infected individuals is six times the number of positive cases reported daily. The colored area represents the variation in the immunity effectiveness due to the variation in the number of asymptomatically infected individuals (4–8 times).

Figure 2 shows the DPC and population-level immunity estimates for Tokyo, Osaka, and Aichi. Considering the range of asymptomatic infections that are estimated to be four to eight times higher than reported DPCs, the numbers also demonstrate the uncertainty in immunity efficacy (Figure 1b). Before the sixth wave, the impact of the infected population on population-level immunity was marginal, but it became relatively dominant thereafter. This trend is also observed in Osaka and Aichi. Therefore, comparable levels of population-level immunity were required in the three prefectures during the fifth to seventh waves.

**Figure 3:**
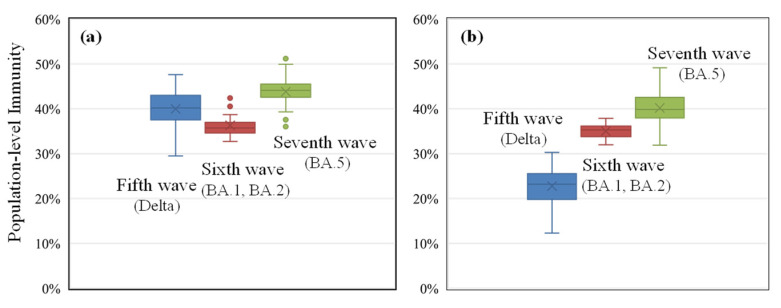
Estimated population-level immunity (obtained by vaccination and infection) for protection against symptomatic infection: (**a**) entire population and (**b**) population aged 10–64 years. The numbers of infected asymptomatic individuals were assumed to be six times that of reported daily positive cases.

To gain additional insight into the relationship between population-level immunity and the number of positive cases, Figure 3 shows the variation in population-level immunity across the 47 prefectures for the entire population and for individuals aged 10–64 years at the peak of the DPCs. For all waves, the required level of population-level immunity for suppression was approximately 40%. The sixth and seventh waves showed a gradual increase in immunity, despite the fact that immunity for the entire population is quite close. Especially for the fifth wave, the immunity of the population aged 10–64 years required for peak formation is less than that of the entire population. The reason is attributable to the strategy of early vaccination campaigns, wherein the older population was prioritized (see Section 1). The fully vaccinated people aged 10–64 years were approximately 20% at the peak of the fifth wave, whereas those aged ≥65 years were over 80% [30].

### 3.3. Confirmed Cases and Vaccination Rates

**Figure 4:**
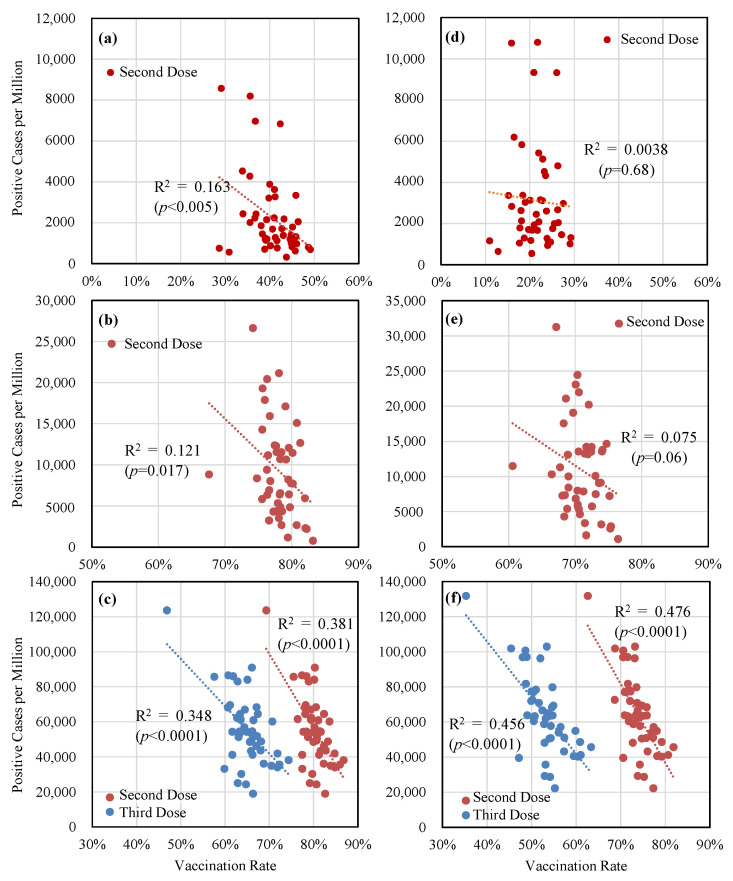
Dependency of confirmed cases per population on the vaccination rates of the second and third doses for (**a**–**c**) the total population and (**d**–**f**) the population aged 10–64 years when the peak of the DPCs was observed in the (**a**,**d**) fifth; (**b**,**e**) sixth; and (**d**,**f**) seventh waves.

Figure 4 depicts the relationship between the number of confirmed cases per population and the vaccination rates for the second and third doses, when the peak of the DPCs was observed. According to the results, a weak inverse correlation was observed between confirmed cases in the fifth and sixth waves, whereas an inverse correlation was observed in the seventh wave.

### 3.4. Verification and Interpolation with Machine Learning

**Figure 5:**
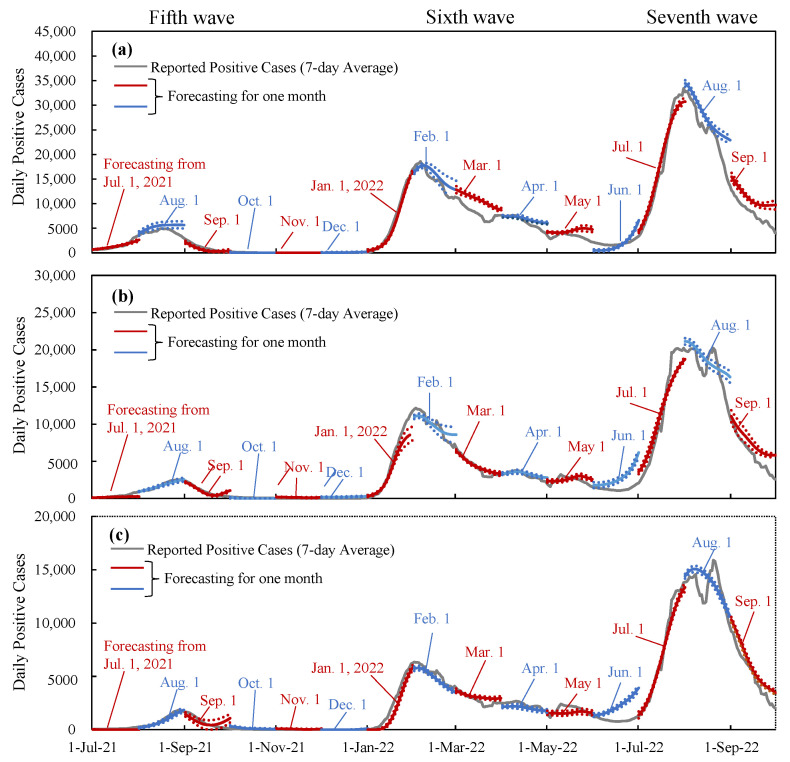
Performance of the prediction of daily positive cases in (**a**) Tokyo, (**b**) Osaka, and (**c**) Aichi. In the forecasting, Twitter and mobility were assumed to be known from the reported values, and the estimated population-level immunity in Figure 2 was used. The dotted lines represent 95% CI.

Using the estimated effectiveness of immunity, we replicated new daily cases using machine learning. In this replication, we assumed future mobility and the number of tweets to be known variables. Figure 5 illustrates forecast examples for one month in Tokyo, Osaka, and Aichi, consisting of 15 time frames. The mean absolute percentage error for the forecast after one month was found to be 23.7% in Tokyo, 28.1% in Osaka, and 27.3% in Aichi for days with more than 500 DPCs.

**Figure 6:**
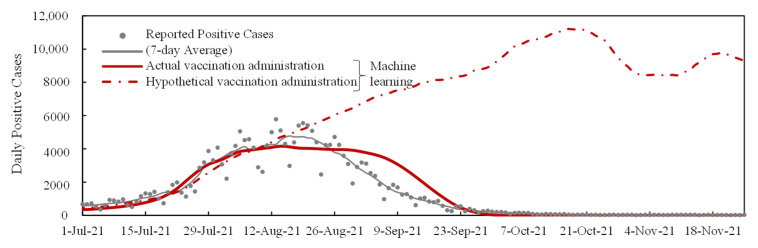
Forecasting daily positive cases in Tokyo based on actual vaccination administration (solid line) and a hypothetical scenario assuming vaccination is not administered after 11 July 2021 (dashed line).

From these results, machine learning was confirmed to be useful for assessing the impact of the number of tweets, mobility, and VE on new DPCs. As a hypothetical scenario, the new vaccine had not been administrated after 11 July 2021. As shown in Figure 6, a further increase in the number of DPCs was expected without vaccination.

## 4. Discussion

This study evaluated the population-level immunity required for transient suppression for Japan’s fifth, sixth, and seventh waves, when the Delta, Omicron variants, and the BA.5 subvariant were predominant, respectively.

**Figure A2:**
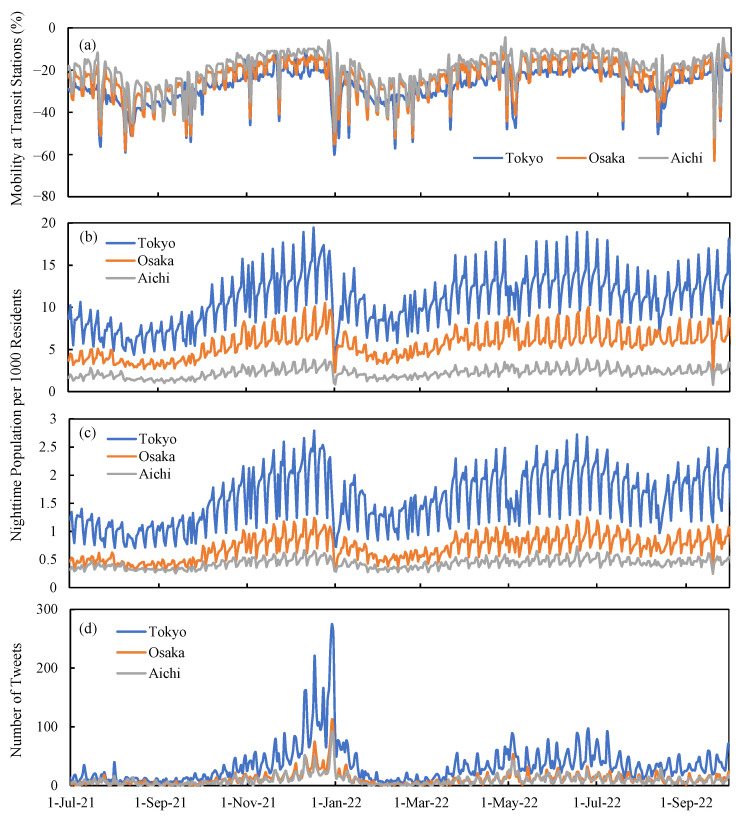
Time series of (**a**) mobility at transit stations and (**b**,**c**) night population in the downtown area, wherein (**b**) 15–64 years old and (**c**) over 65 years old. (**d**) The number of tweets associated with risk (social drinking and barbecue events) is also shown. The number of older people in the downtown area is much lower than that of young people and transmission is suppressed compared with the mobility at the transit station.

**Figure A3:**
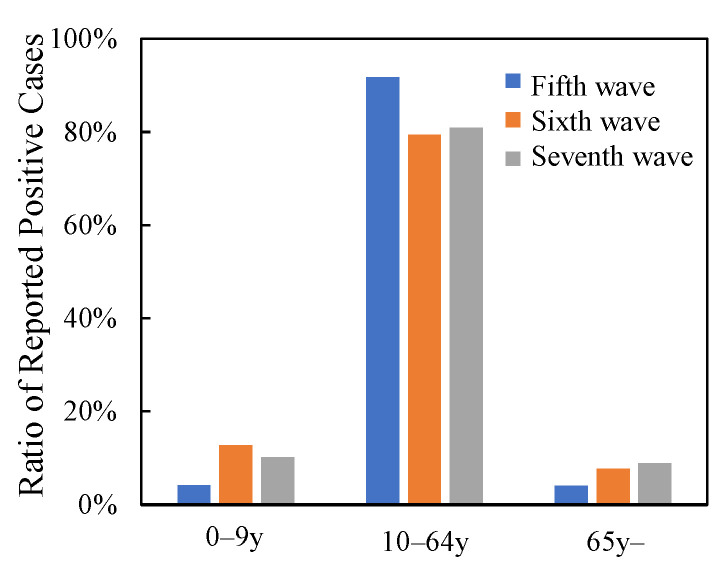
Total reported positive cases by age group in Tokyo.

In the fifth wave, the population-level immunity required to suppress the spread of infection was 40% and 20% for the entire population and the population aged 10–64 years, respectively. As shown in Figure A3, most cases were found for the population aged 10–64 years; therefore, an immunity of 20% would be close to the immunity required in reality. Unlike the later wave, the mobility for the population aged 10–64 years was smaller in the fifth wave than in the later waves due to the declaration of the state of emergency. The reduction in mobility in downtown areas was approximately 40%, 25%, and 20% lower than the levels in Tokyo, Osaka, and Aichi, respectively (see Figure A2). When social activity is low, the transmission of SARS-CoV-2 may also be suppressed. The proportion of the population aged 10–64 years who contracted the infection was 91%, 79%, and 81% in the fifth, sixth, and seventh waves, respectively (Figure A3); therefore, this metric would be closely related to the number of cases.

The variability in population-level immunity required for suppression during the fifth wave was greater than for the two remaining waves. The reasons for this tendency are (1) the smaller transmissibility than the following variants [38] and the longer generation time (5–8 days for the Delta variant) [39] than those of the Omicron variants (2–3 days) [40], and (2) the high ratio of serious and death cases to reported cases for the Delta variant; therefore, population mobility and activity decreased more than those of subsequent waves, when the Omicron variant was dominant (e.g., −36%, −34%, and −25% for the fifth, sixth, and seventh waves in Tokyo, respectively).

During the sixth wave, 9.5% of the population was infected assuming that the number of people with asymptotic infection was six times (Section 2.3) greater than the population with symptomatic infection in Japan. The population level of immunity required for peak formation was relatively consistent across the 47 prefectures. The quasi state of emergency was administered during this wave, while the mobility data suggested that the population was more active than during the fifth wave (Figure A2). This tendency was also confirmed by the number of tweets (Figure A2d).

During the seventh wave, the proportion of immunity obtained through infection and vaccination was nearly equivalent. Given the unreported number of asymptomatic infections, approximately 29% of the population would be infected based on the proportion of confirmed cases per population which was 4.1%. This value was greater than the 32% (CI: 27–37%) value reported for the N protein, a metric of infection [41] in Tokyo [42]. This information was obtained from blood donation participants. Multiple factors could explain this difference. First, a factor for estimating the number of positive cases without symptoms was determined by the survey test conducted in the downtown areas, whereas the group for the N protein test was for the participants who donated blood. Additionally, the antibody obtained through infection without symptoms was weaker and its duration was brief [42], as measured in November 2022 which was three months after the peak of the seventh wave. The percentage reported of the population with the N protein in Osaka and Aichi was 41% (CI: 34–47%) and 28% (CI: 22–33%), respectively [42], whereas the estimated ratio of the infected population was 44% and 35%, respectively.

As shown in Figure 4, the correlation between confirmed cases per population and the vaccination rate was significant for the entire population, while that for the population aged 10–64 years was significant only during the seventh wave. As shown in Figure 1, even in this wave, vaccination-based immunity remained at 20% or higher. The weak correlation observed in earlier waves may have been due to the (quasi) state of emergency policies implemented during those waves and the fact that only a small portion of the population was infected at the time. As shown in Appendix B, the authors of this study also observed a significant correlation at the conclusion of the seventh wave.

To validate the aforementioned heuristic method, we used machine learning to estimate the DPC. As depicted in Figure 5, the prediction based on the assumption that future behavioral and vaccination parameters are known was in good agreement with the cases reported by the three prefectures. This comparison suggested that the heuristic method based on the numerical derivation of immunity was appropriate. Note that our vaccination parameters and the prediction model have been verified to be acceptable using machine learning under various periods during the COVID-19 pandemic [18,23,28,32,43]. Using machine learning, we have provided an interpolation of what would have happened if the vaccination was not administrated in Tokyo after 11 July 2021, during the Olympic Games (Figure 6). From the computational example, the decay of the wave was mainly attributable to vaccination. Without vaccination, the wave would continue to increase for a while even in a state of emergency after the Olympic Games. Our result was consistent with a previous study, suggesting that the DPC may increase during and after the Olympic Games [43]. Note that in that study [42] the main emphasis was on vaccination planning, and thus a straightforward comparison cannot be made. Additionally, the input parameters used in this study, such as the number of tweets, are different from other studies.

The primary limitations of this study are as follows:

1. Due to the limited number of people who were infected in Japan prior to the sixth wave, the population with asymptomatic infection is a major source of uncertainty (see Figure 2). Even when considering a factor of four to eight for the ratio of asymptomatic infected individuals to the DPC, the population-level immunity in the sixth and seventh waves varied by 8% and 12%, respectively. Consequently, the uncertainty associated with the box plots in Figure 3 would be somewhat greater.
2. There is additional uncertainty in the definition of waves, and the number of DPCs per population in the fifth wave is lower than in previous waves, making the definition of the wave relatively arbitrary in certain prefectures with small populations (for example, the maximum DPC in the Tottori prefecture was 47). In the interest of simplification, it is assumed that the decay of infection-acquired immunity is identical to that of vaccination. It is known that the former is longer than the latter. This simplification does not lead to significant differences as the number of infected people in Japan was limited until the wave we focused on. Similarly, the consideration of hybrid immunity [44,45,46], defined as immunity acquired through complete vaccination and infection, was marginal.
3. Note that even for the abovementioned limitations, its impact on the forecasting or the transmission was demonstrated to be marginal through machine learning replication.

## 5. Conclusions

This study numerically discussed the population-level immunity required for transient suppression across the fifth, sixth, and seventh waves of COVID-19 across the 47 prefectures in Japan. A feature of COVID-19 infection in Japan was that only 11% of the population was infected before the seventh wave. Therefore, the uncertainty factor associated with immunity (such as hybrid immunity and reinfection) was relatively small. The population-level immunity required for transient suppression was consistent across prefectures. The effect of the vaccination rate on positive cases per population was mild for the fifth and sixth waves, when the effect of the restriction was dominant. The correlation between the vaccination ratio and positive cases was more statistically significant for the seventh wave. Meanwhile, viral infectivity and population activity that increased with subsequent waves characterize the immunity required for suppression at the population level. Our discussion suggested that if viral infectivity is known, then the number of people who can become infected before transient suppression can be roughly estimated. This hypothesis can be confirmed using machine learning. The combination of heuristic derivation and machine learning would be beneficial for policy formulation and the allocation of medical resources, particularly for vaccination strategies.

## Figures and Tables

**Table 1:** Period for the fifth to seventh waves in all Japanese prefectures.

		Fifth Wave		Sixth Wave		Seventh Wave	
No.	Prefecture	Start	Peak	Start	Peak	Start	Peak
1	Hokkaido	30-Jun-21	21-Aug-21	15-Jan-22	8-Feb-22	28-Jun-22	21-Aug-22
2	Aomori	1-Aug-21	29-Aug-21	16-Nov-21	8-Mar-22	10-Jun-22	27-Aug-22
3	Iwate	20-Jun-21	17-Aug-21	14-Oct-21	28-Feb-22	25-Jun-22	21-Aug-22
4	Miyagi	31-Jul-21	20-Aug-21	7-Jan-22	18-Feb-22	27-Jun-22	21-Aug-22
5	Akita	8-Aug-21	22-Aug-21	25-Jan-22	27-Jan-22	29-Jun-22	21-Aug-22
6	Yamagata	21-Jul-21	20-Aug-21	26-Jan-22	4-Feb-22	22-Jun-22	21-Aug-22
7	Fukushima	10-Jul-21	15-Aug-21	26-Jan-22	5-Feb-22	28-Jun-22	21-Aug-22
8	Ibaraki	2-Jul-21	20-Aug-21	17-Jan-22	9-Feb-22	11-Jun-22	22-Aug-22
9	Tochigi	29-Jun-21	22-Aug-21	27-Dec-21	17-Feb-22	7-Jul-22	2-Aug-22
10	Gumma	22-Jul-21	21-Aug-21	18-Jan-22	2-Feb-22	26-Jun-22	21-Aug-22
11	Saitama	8-Jul-21	18-Aug-21	17-Jan-22	8-Feb-22	2-Jul-22	3-Aug-22
12	Chiba	9-Jul-21	18-Aug-21	16-Jan-22	7-Feb-22	5-Jul-22	31-Jul-22
13	Tokyo	25-Jun-21	16-Aug-21	17-Jan-22	5-Feb-22	30-Jun-22	31-Jul-22
14	Kanagawa	24-Jun-21	23-Aug-21	21-Jan-22	7-Feb-22	1-Jul-22	30-Jul-22
15	Niigata	29-Jun-21	24-Aug-21	16-Jan-22	27-Jan-22	26-Jun-22	21-Aug-22
16	Toyama	28-Jul-21	19-Aug-21	6-Jan-22	1-Mar-22	28-Jun-22	21-Aug-22
17	Ishikawa	13-Jul-21	29-Jul-21	22-Jan-22	3-Feb-22	27-Jun-22	22-Aug-22
18	Fukui	7-Jun-21	27-Aug-21	6-Dec-21	10-Mar-22	30-Jun-22	22-Aug-22
19	Yamanashi	16-Jul-21	20-Aug-21	21-Jan-22	28-Jan-22	18-Jun-22	20-Aug-22
20	Nagano	27-Jul-21	21-Aug-21	10-Jan-22	3-Feb-22	25-Jun-22	21-Aug-22
21	Gifu	9-Aug-21	27-Aug-21	22-Dec-21	16-Feb-22	21-Jun-22	20-Aug-22
22	Shizuoka	30-Jul-21	22-Aug-21	12-Jan-22	7-Feb-22	19-Jun-22	21-Aug-22
23	Aichi	8-Aug-21	29-Aug-21	21-Dec-21	16-Feb-22	17-Jun-22	20-Aug-22
24	Mie	14-Aug-21	24-Aug-21	13-Jan-22	6-Feb-22	13-Jun-22	21-Aug-22
25	Shiga	25-Jul-21	22-Aug-21	11-Jan-22	7-Feb-22	15-Jun-22	22-Aug-22
26	Kyoto	17-Jul-21	23-Aug-21	12-Jan-22	7-Feb-22	2-Jul-22	2-Aug-22
27	Osaka	9-Jul-21	29-Aug-21	9-Jan-22	8-Feb-22	18-Jun-22	20-Aug-22
28	Hyogo	20-Jul-21	25-Aug-21	13-Jan-22	7-Feb-22	15-Jun-22	21-Aug-22
29	Nara	10-Jul-21	29-Aug-21	3-Jan-22	17-Feb-22	23-Jun-22	22-Aug-22
30	Wakayama	16-Jul-21	22-Aug-21	15-Jan-22	5-Feb-22	15-Jun-22	21-Aug-22
31	Tottori	9-Jul-21	2-Aug-21	28-Nov-22	24-Feb-22	13-Jun-22	20-Aug-22
32	Shimane	24-Jun-21	27-Aug-21	15-Jan-22	20-Jan-22	13-Jun-22	20-Aug-22
33	Okayama	26-Jul-21	20-Aug-21	15-Jan-22	3-Feb-22	26-Jun-22	21-Aug-22
34	Hiroshima	6-Aug-21	22-Aug-21	8-Jan-22	23-Jan-22	24-Jun-22	22-Aug-22
35	Yamaguchi	5-Aug-21	21-Aug-21	3-Jan-22	27-Jan-22	21-Jun-22	21-Aug-22
36	Tokushima	14-Aug-21	27-Aug-21	2-Jan-22	1-Mar-22	18-Jul-22	23-Aug-22
37	Kagawa	27-Jul-21	22-Aug-21	31-Dec-21	20-Feb-22	24-Jun-22	20-Aug-22
38	Ehime	29-Jul-21	19-Aug-21	10-Jan-22	31-Jan-22	15-Jun-22	20-Aug-22
39	Kochi	16-Aug-21	24-Aug-21	19-Jan-22	12-Feb-22	19-Jun-22	24-Aug-22
40	Fukuoka	12-Jul-21	21-Aug-21	13-Jan-22	5-Feb-22	15-Jun-22	20-Aug-22
41	Saga	9-Aug-21	20-Aug-21	14-Jan-22	5-Feb-22	5-Jun-22	20-Aug-22
42	Nagasaki	18-Jul-21	20-Aug-21	18-Jan-22	30-Jan-22	1-Jul-22	21-Aug-22
43	Kumamoto	23-Jul-21	20-Aug-21	17-Jan-22	30-Jan-22	6-Jun-22	20-Aug-22
44	Oita	12-Aug-21	22-Aug-21	19-Jan-22	1-Feb-22	23-Jun-22	20-Aug-22
45	Miyazaki	6-Aug-21	24-Aug-21	20-Jan-22	29-Jan-22	19-Jun-22	21-Aug-22
46	Kagoshima	8-Aug-21	19-Aug-21	17-Jan-22	4-Feb-22	14-Jun-22	21-Aug-22
47	Okinawa	8-Jul-21	17-Aug-21	7-Jan-22	15-Jan-22	14-May-22	2-Aug-22

**Table 2:** Population, population density, and the amount of reported positive cases reported from the start to the peak for the fifth to seventh waves in all Japanese prefectures.

				Total Positive Cases		
No.	Prefecture	Population	Population Density (per km^2^)	Fifth Wave	Sixth Wave	Seventh Wave
1	Hokkaido	5,226,603	62.6	11,297	55,724	215,433
2	Aomori	1,259,615	130.6	1519	19,016	75,803
3	Iwate	1,220,823	79.9	865	7268	41,598
4	Miyagi	2,281,989	332.6	2835	17,700	105,508
5	Akita	971,288	83.5	318	753	37,124
6	Yamagata	1,069,562	160.8	668	2358	38,085
7	Fukushima	1,862,059	135.1	2243	4989	65,197
8	Ibaraki	2,907,675	477.0	7073	22,438	126,973
9	Tochigi	1,955,401	305.1	4778	22,427	37,213
10	Gumma	1,958,101	307.8	4393	12,542	85,080
11	Saitama	7,393,799	1962.3	33,513	91,036	224,180
12	Chiba	6,322,892	1244.2	27,062	72,937	153,762
13	Tokyo	13,843,329	6579.5	113,536	267,383	571,349
14	Kanagawa	9,220,206	3816.3	64,261	114,260	230,852
15	Niigata	2,213,174	213.5	2634	5089	92,893
16	Toyama	1,047,674	512.1	1204	13,287	51,134
17	Ishikawa	1,132,656	270.6	859	4963	61,562
18	Fukui	774,583	184.9	1389	13,260	47,221
19	Yamanashi	820,997	195.4	1525	2195	40,526
20	Nagano	2,071,737	158.1	1826	10,061	74,963
21	Gifu	2,016,791	206.5	4413	24,353	110,110
22	Shizuoka	3,686,260	508.1	7422	30,258	200,497
23	Aichi	7,558,802	1477.5	24,286	154,567	515,468
24	Mie	1,800,557	312.5	3058	11,824	102,564
25	Shiga	1,418,843	376.7	3170	16,395	88,404
26	Kyoto	2,530,542	548.6	9177	45,319	84,156
27	Osaka	8,839,511	4649.9	60,432	235,649	757,601
28	Hyogo	5,523,625	657.9	18,102	88,104	384,344
29	Nara	1,344,739	364.3	4498	28,476	82,196
30	Wakayama	944,432	199.8	1254	7908	58,189
31	Tottori	556,788	158.8	319	4474	29,649
32	Shimane	672,815	100.3	678	782	43,418
33	Okayama	1,893,791	270.2	3247	13,098	97,163
34	Hiroshima	2,812,433	331.7	3401	16,441	152,939
35	Yamaguchi	1,356,110	221.8	943	5900	70,437
36	Tokushima	734,949	177.2	593	7867	35,718
37	Kagawa	973,896	523.0	1420	10,903	53,050
38	Ehime	1,356,219	238.8	1053	4793	72,963
39	Kochi	701,167	98.7	580	4458	45,237
40	Fukuoka	5,124,170	1057.2	19,918	73,264	443,370
41	Saga	818,222	335.3	1156	7706	70,347
42	Nagasaki	1,335,938	325.4	1306	6526	91,356
43	Kumamoto	1,758,645	242.0	3616	11,233	159,905
44	Oita	1,141,741	223.9	1585	4928	76,497
45	Miyazaki	1,087,241	160.0	1402	3537	90,348
46	Kagoshima	1,617,517	178.8	1824	8681	135,958
47	Okinawa	1,485,118	652.2	12,733	13,124	183,673

## Data Availability

Not applicable.

## Supplementary Materials

The following supporting information can be downloaded at: https://www.mdpi.com/article/10.3390/vaccines11091457/s1, Video S1: Time course of confirmed cases per population on the vaccination rates for the total population.

## Appendix A

Figure A1 depicts the total number of positive cases per million per prefecture as of 30 September 2022. Figure A1’s numbers correspond with those in Table 2.

It has been extensively discussed that behavior, including mobility, is closely related to infection. In this study, two metrics were considered the assessments for social behavior: (i) mobility at the transit stations and (ii) nighttime population in the downtown area, including restaurants and bars [47].

Mobility data were obtained from the COVID-19 community mobility reports reported by Google [48]. The mobility data were categorized into six types by location, with mobility at transit stations being often used as a metric for viral transmission.

The nighttime population who stayed in the downtown area near restaurants and bars was obtained from NTT DOCOMO, INC, also accessible under the project of the Cabinet Secretariat COVID-19 AI Simulation Project, Japan. The dataset contains information about ties, such as age (5-year age groups) and gender, and in this case, we used the population residing in the downtown area at 21:00. Figure A2 depicts the time courses of these datasets. Figure A2 also depicts the number of tweets (barbecue events and social drinking) in three prefectures. The machine learning prediction utilized these parameters.

Figure A3 shows each age category as a percentage of the total number of positive cases from the start date of a wave to the date when the highest number of positive cases was observed in Tokyo. The start dates of a wave and the peak dates observed in the peak positive cases correspond to Table 1.

## Appendix B

Corresponding to Figure 4, the time course of confirmed cases per population on the vaccination rates for the total population is shown in the animations in the Video S1.

## References

[1] World Health Organization (WHO) Coronavius (COVID-19) Dashboard. https://covid19.who.int/.

[2] Badr H.S., Du H., Marshall M., Dong E., Squire M.M., Gardner L.M. (2020). Association between mobility patterns and COVID-19 transmission in the USA: A mathematical modelling study. Lancet Infect. Dis..

[3] Kodera S., Hikita K., Rashed E.A., Hirata A. (2022). The effects of time window-averaged mobility on effective reproduction number of COVID-19 viral variants in urban cities. J. Urban Health.

[4] Shaw R., Kim Y., Hua J. (2020). Governance, technology and citizen behavior in pandemic: Lessons from COVID-19 in East Asia. Prog. Disaster Sci..

[5] Han E., Tan M.M.J., Turk E., Sridhar D., Leung G.M., Shibuya K., Asgari N., Oh J., García-Basteiro A.L., Hanefeld J. (2020). Lessons learnt from easing COVID-19 restrictions: An analysis of countries and regions in Asia Pacific and Europe. Lancet.

[6] Unruh L., Allin S., Marchildon G., Burke S., Barry S., Siersbaek R., Thomas S., Rajan S., Koval A., Alexander M. (2022). A comparison of 2020 health policy responses to the COVID-19 pandemic in Canada, Ireland, the United Kingdom and the United States of America. Health Policy.

[7] Diao Y., Kodera S., Anzai D., Gomez-Tames J., Rashed E.A., Hirata A. (2021). Influence of population density, temperature, and absolute humidity on spread and decay durations of COVID-19: A comparative study of scenarios in China, England, Germany, and Japan. One Health.

[8] Sun Z., Zhang H., Yang Y., Wan H., Wang Y. (2020). Impacts of geographic factors and population density on the COVID-19 spreading under the lockdown policies of China. Sci. Total Environ..

[9] Lau H., Khosrawipour V., Kocbach P., Mikolajczyk A., Schubert J., Bania J., Khosrawipour T. (2020). The positive impact of lockdown in Wuhan on containing the COVID-19 outbreak in China. J. Travel Med..

[10] Odagaki T. (2020). Analysis of the outbreak of COVID-19 in Japan by SIQR model. Infect. Dis. Model..

[11] Tetteh J.N.A., Nguyen V.K., Hernandez-Vargas E.A. (2021). Network models to evaluate vaccine strategies towards herd immunity in COVID-19. J. Theor. Biol..

[12] Tang J.W., Wu S., Kwok K.O. (2021). Can Asia now learn from the experience of the West?. Clin. Microbiol. Infect..

[13] Tkachenko A.V., Maslov S., Elbanna A., Wong G.N., Weiner Z.J., Goldenfeld N. (2021). Time-dependent heterogeneity leads to transient suppression of the COVID-19 epidemic, not herd immunity. Proc. Natl. Acad. Sci. USA.

[14] Global Change Data Lab Our World in Data. https://ourworldindata.org/.

[15] Arashiro T., Arima Y., Muraoka H., Sato A., Oba K., Uehara Y., Arioka H., Yanai H., Kuramochi J., Ihara G. (2023). COVID-19 Vaccine Effectiveness Against Symptomatic SARS-CoV-2 Infection During Delta-Dominant and Omicron-Dominant Periods in Japan: A Multicenter Prospective Case-control Study (Factors Associated with SARS-CoV-2 Infection and the Effectiveness of COVID-19 Vaccines Study). Clin. Infect. Dis..

[16] Buchan S.A., Chung H., Brown K.A., Austin P.C., Fell D.B., Gubbay J.B., Nasreen S., Schwartz K.L., Sundaram M.E., Tadrous M. (2022). Estimated effectiveness of COVID-19 vaccines against Omicron or Delta symptomatic infection and severe outcomes. JAMA Netw. Open.

[17] Reis B.Y., Barda N., Leshchinsky M., Kepten E., Hernán M.A., Lipsitch M., Dagan N., Balicer R.D. (2021). Effectiveness of BNT162b2 vaccine against Delta variant in adolescents. N. Engl. J. Med..

[18] Kodera S., Rashed E.A., Hirata A. (2022). Estimation of real-world vaccination effectiveness of mRNA COVID-19 vaccines against Delta and Omicron variants in Japan. Vaccines.

[19] Andrews N., Stowe J., Kirsebom F., Toffa S., Rickeard T., Gallagher E., Gower C., Kall M., Groves N., O’Connell A.-M. (2022). COVID-19 vaccine effectiveness against the Omicron (B.1.1.529) variant. N. Engl. J. Med..

[20] Kirsebom F.C.M., Andrews N., Stowe J., Toffa S., Sachdeva R., Gallagher E., Groves N., O’Connell A.-M., Chand M., Ramsay M. (2022). COVID-19 vaccine effectiveness against the omicron (BA.2) variant in England. Lancet Infect. Dis..

[21] Seong H., Hyun H.J., Yun J.G., Noh J.Y., Cheong H.J., Kim W.J., Song J.Y. (2021). Comparison of the second and third waves of the COVID-19 pandemic in South Korea: Importance of early public health intervention. Int. J. Infect. Dis..

[22] Araf Y., Akter F., Tang Y., Fatemi R., Parvez M.S.A., Zheng C., Hossain M.G. (2022). Omicron variant of SARS-CoV-2: Genomics, transmissibility, and responses to current COVID-19 vaccines. J. Med. Virol..

[23] Kodera S., Niimi Y., Rashed E.A., Yoshinaga N., Toyoda M., Hirata A. (2022). Estimation of mRNA COVID-19 vaccination effectiveness in Tokyo for Omicron variants BA.2 and BA.5: Effect of social behavior. Vaccines.

[24] Ministry of Health Labour and Welfare Visualizing the Data: Information on COVID-19 Infections. https://covid19.mhlw.go.jp/en/.

[25] Sanada T., Honda T., Yasui F., Yamaji K., Munakata T., Yamamoto N., Kurano M., Matsumoto Y., Kohno R., Toyama S. (2022). Serologic survey of IgG against SARS-CoV-2 among hospital visitors without a history of SARS-CoV-2 Infection in Tokyo, 2020–2021. J. Epidemiol..

[26] Randolph H.E., Barreiro L.B. (2020). Herd Immunity: Understanding COVID-19. Immunity.

[27] Núñez-Zapata S.F., Benites-Peralta B., Mayta-Tristan P., Rodríguez-Morales A.J. (2021). High seroprevalence for SARS-CoV-2 infection in South America, but still not enough for herd immunity!. Int. J. Infect. Dis..

[28] Rashed E.A., Kodera S., Hirata A. (2022). COVID-19 forecasting using new viral variants and vaccination effectiveness models. Comput. Biol. Med..

[29] Ministry of Health Labour and Welfare Open Data (in Japanese). https://www.mhlw.go.jp/stf/covid-19/open-data.html.

[30] Digital Agency Vaccination Record System. https://info.vrs.digital.go.jp/opendata/.

[31] Khoury D.S., Cromer D., Reynaldi A., Schlub T.E., Wheatley A.K., Juno J.A., Subbarao K., Kent S.J., Triccas J.A., Davenport M.P. (2021). Neutralizing antibody levels are highly predictive of immune protection from symptomatic SARS-CoV-2 infection. Nat. Med..

[32] Kodera S., Takada A., Rashed E.A., Hirata A. (2023). Projection of COVID-19 Positive Cases Considering Hybrid Immunity: Case Study in Tokyo. Vaccines.

[33] Tokyo Metropolitan Agent the Disaster Prevention Information, Tokyo. https://www.bousai.metro.tokyo.lg.jp/taisaku/saigai/index.html.

[34] Cooper I., Mondal A., Antonopoulos C.G. (2020). A SIR model assumption for the spread of COVID-19 in different communities. Chaos Solitons Fractals.

[35] Carcione J.M., Santos J.E., Bagaini C., Ba J. (2020). A simulation of a COVID-19 epidemic based on a deterministic SEIR model. Front. Public Health.

[36] Annas S., Isbar Pratama M., Rifandi M., Sanusi W., Side S. (2020). Stability analysis and numerical simulation of SEIR model for pandemic COVID-19 spread in Indonesia. Chaos Solitons Fractals.

[37] Arora P., Kumar H., Panigrahi B.K. (2020). Prediction and analysis of COVID-19 positive cases using deep learning models: A descriptive case study of India. Chaos Solitons Fractals.

[38] Pearson C.A.B., Silal S.P., Li M.W.Z., Dushoff J., Bolker B.M., Abbott S., van Schalkwyk C., Benjamin M. (2021). Bounding the levels of transmissibility & immune evasion of the Omicron variant in South Africa. medRxiv.

[39] Li B., Deng A., Li K., Hu Y., Li Z., Shi Y., Xiong Q., Liu Z., Guo Q., Zou L. (2022). Viral infection and transmission in a large, well-traced outbreak caused by the SARS-CoV-2 Delta variant. Nat. Commun..

[40] Manica M., De Bellis A., Guzzetta G., Mancuso P., Vicentini M., Venturelli F., Zerbini A., Bisaccia E., Litvinova M., Menegale F. (2022). Intrinsic generation time of the SARS-CoV-2 Omicron variant: An observational study of household transmission. Lancet Reg. Health Eur..

[41] Sethuraman N., Jeremiah S.S., Ryo A. (2020). Interpreting diagnostic tests for SARS-CoV-2. JAMA.

[42] Ministry of Health Labour and Welfare Advisory Boad Meetings of COVID-19 Measures. https://www.mhlw.go.jp/stf/seisakunitsuite/bunya/0000121431_00294.html.

[43] Hirata A., Kodera S., Diao Y., Rashed E.A. (2022). Did the Tokyo Olympic Games enhance the transmission of COVID-19? An interpretation with machine learning. Comput. Biol. Med..

[44] Crotty S. (2021). Hybrid immunity. Science.

[45] Sette A., Crotty S. (2022). Immunological memory to SARS-CoV-2 infection and COVID-19 vaccines. Immunol. Rev..

[46] Bhattacharya M., Sharma A.R., Dhama K., Agoramoorthy G., Chakraborty C. (2022). Hybrid immunity against COVID-19 in different countries with a special emphasis on the Indian scenario during the Omicron period. Int. Immunopharmacol..

[47] Nakanishi M., Shibasaki R., Yamasaki S., Miyazawa S., Usami S., Nishiura H., Nishida A. (2021). On-site dining in Tokyo during the COVID-19 pandemic: Time series analysis using mobile phone location data. JMIR Mhealth Uhealth.

[48] Google COVID-19 Community Mobility Reports. https://www.google.com/covid19/mobility/.

